# Integrated Management of Complex Interplay Between Severe Enduring Mental Illness and Type I Diabetes on an Acute Psychiatric Ward: A Case Report

**DOI:** 10.1192/bjo.2026.11892

**Published:** 2026-06-30

**Authors:** Kotryna Ryan-Gilbank, Omer Malik, Betsy Allbright, Racheal Amankwaa, Raluca Miutescu

**Affiliations:** Cygnet Health Care, London, United Kingdom

## Abstract

**Aims:**

Patients with severe mental illness frequently present with complex physical health comorbidities, creating significant challenges for safe inpatient management on acute psychiatric wards. Patient’s impaired capacity and behavioural disturbance can further complicate engagement with essential medical care. This case highlights the importance of integrated physical and mental health management and multidisciplinary team collaboration in achieving positive outcomes.

**Methods:**

X is a 46-year-old female with a diagnosis of Schizoaffective disorder and poorly controlled Type I Diabetes. She presented with a progressive deterioration in mental state following discontinuation of Clozapine and Lithium. Intermittent refusal of BG monitoring, erratic and unhealthy dietary habits, and variable cooperation with insulin administration led to significant glycaemic instability, requiring coordinated Multidisciplinary team (MDT) input to ensure patient safety. Patient was transitioned to Paliperidone depot injection due to the favourable metabolic profile.

A coordinated MDT approach was implemented. Nursing staff adhered to a structured rapid-acting insulin management plan, administering additional doses in response to BG readings. A Freestyle Libre continuous glucose monitoring sensor was introduced, with staff educating the patient, helping to set up alerts for hypo- and hyperglycaemia. Dietitian’s input was provided, alongside liaison with hospital catering services to facilitate healthier food substitutions.

**Results:**

As a result the patient’s glycaemic control improved markedly, with reduced BG fluctuations and decreased need for additional rapid-acting insulin. This improvement was reflected in a significant reduction in HbA1c from 92 mmol/mol (21/11/25) to 69 mmol/mol (14/01/26) within less than 3 months. The patient demonstrated sustained behavioural change, discontinuing takeaway food and adopting healthier eating habits.

Symptom severity, measured using the Brief Psychiatric Rating Scale (BPRS), reduced from 52 to 29, representing 44 % demonstrating a substantial reduction in psychotic, affective, and behavioural symptoms.

Occupational therapy task observation scale (OTTOS) total score increased from 61/200 to 190/200, representing 64.5% increase, indicating a substantial improvement in both task performance and overall functional behaviour.

GAP (global assessment of progress) score improved from 40/70 on admission to 59/70, representing an absolute increase of 19 points and reflecting a 27% improvement across the different domains, highlighting a marked improvement in clinical stability and functional engagement.

**Conclusion:**

Integrated physical and mental health care is essential for patients with severe mental illness and complex comorbidities. This case demonstrates that even during acute psychiatric deterioration, effective management of complex physical health conditions is achievable through structured care plans, consistent nursing input, and strong multidisciplinary team collaboration.

